# Intervention with EMDR on a sample of healthcare workers in the nephrology and dialysis service during the COVID-19 emergency: from immediate treatment effect to long-term maintenance

**DOI:** 10.3389/fpsyg.2023.1120203

**Published:** 2023-05-09

**Authors:** Caterina Belvedere, Paolo Fabbrini, Elena Alberghini, Simona Anna Ghedini, Isabel Fernandez, Giada Maslovaric, Marco Pagani, Eugenio Gallina

**Affiliations:** ^1^ASST Nord Milano, Milan, Italy; ^2^EMDR Italy Association, Varedo, Italy; ^3^Ospedale Bassini, Milan, Lombardy, Italy; ^4^EMDR Europe, Schaffhausen, Switzerland; ^5^Institute of Cognitive Sciences and Technologies, Consiglio Nazionale delle Ricerche, Rome, Italy; ^6^Centro di Ricerca e Studi in Psicotraumatologia (CRSP), Milano, Italy

**Keywords:** EMDR, COVID-19, nephrology, healthcare workers, mental health, group treatment, psychological support

## Abstract

**Background:**

During the COVID-19 pandemic, psychological support was provided to healthcare workers in Nephrology and Dialysis Operative Unit of the Azienda Ospedaliera Bassini using an EMDR group protocol to decrease posttraumatic stress symptoms in the medium and long term. The aim of this study was to demonstrate the effectiveness of EMDR treatment to reduce post-traumatic stress symptoms at the end of the first pandemic wave and its progress over time in the subsequent phases of the health emergency.

**Methods:**

The sample of study consisted of 43 healthcare workers from the Nephrology and Dialysis Service who spontaneously decided to take part in the Brief EMDR treatment. Statistical analyses were carried out to compare the data collected with the IES-R, the Emotion Thermometer and the Post-Traumatic Growth Scale. The comparisons covered pre-treatment, post-treatment and follow-up.

**Results:**

The results show a significant clinical improvement in reducing PTSD symptoms following the Brief EMDR group treatment. The comparison between PRE and POST treatment (DELTA1) regarding the scores from IES-R and Emotion Thermometer, highlighted the important statistically change that occurred in terms of symptomatology reduction (*p* < 0.001). By comparing POST and FU (DELTA2), it was observed that all variables except avoidance show a significant weakening of the effect with time (*p* < 0.001), but the magnitude of this effect is much smaller than the improvement found in DELTA1. DELTA 3 analysis finally made it possible to highlight how the treatment effect is maintained almost intact at follow-up. In fact, the maintenance of a better situation at follow-up was observed, in the course of re-traumatization linked to the new wave, compared to the initial data (*p* < 0.001).

**Conclusion:**

The COVID-19 health emergency has significantly impacted hospital healthcare workers, leading to a high risk of developing PTSD symptoms. A psychological intervention aimed at the operators themselves is therefore of great importance.

## Introduction

Protecting the mental health of staff members is considered a fundamental goal (World Health Organization, 2020). Targeted psychological support interventions lead to preventing the development of mental disorders (Matsuishi et al., 2012) or burn-out syndrome (Giusti et al., 2020), preserving staff and also reducing the economic and sustainability impact of the entire system (Campion et al., 2020). Failure to provide adequate psychological treatment to people involved in a critical event such as the one in question would be to ignore their needs (Dyregorov and Yule, 2008).

In the course of any emergency, different types of victims can be distinguished (Taylor and Frazer, 1981; Iacolino and Cervellione, 2019). Medical personnel are not only among the victims who provide assistance, but can become victims on several levels. Besides direct traumatization, in fact, there is also vicarious traumatization linked to continuous exposure to traumatized subjects (Brady et al., 1999; Sinclair and Hamill, 2007). Although healthcare workers tend to develop a greater tolerance to highly stressful or traumatic events, as they are used to dealing with critical health situations, the COVID event is an extraordinary situation due to the continuous exposure, lack of prior knowledge, frequent failures, sudden deaths of patients far from their loved ones, isolation, not knowing when the emergency will end, etc. All this can lead to even long-term effects on physical and mental health.

During the first wave of the COVID-19 emergency, the Bassini hospital in Cinisello Balsamo was rapidly converted into an entirely COVID-positive facility. As a consequence, the Nephrology Department of the O.U. of Nephrology and Dialysis also responded to the need to offer beds and treatment to COVID patients, temporarily losing its specificity in the treatment of nephropathies. The Service is composed of different units, namely the Nephrology and Dialysis Department in Cinisello Balsamo, and the CAL (Centro di Assistenza Limitata) in Sesto S. Giovanni. The fragmentation of the operating unit, not only because of the different geographical location, but also because of the different tasks involved in taking care of patients, has led to the establishment of distinct realities with respect to the presence of the SARS-CoV-2 virus: a ‘dirty’ area, that of the Department, and a ‘clean’ area, that of the two Dialysis Units. However, even the clean area often received suspicious patients (whose positivity was later confirmed in numerous cases). In addition, the nurses of the clean areas, as trained dialysis caregivers, were often instructed to go to COVID wards to dialyze positive in-patients.

Several studies carried out in the context of previous medical emergencies (Chan, 2004; Lee et al., 2007, 2018; Lin et al., 2007) have highlighted the frequent development of post-traumatic symptoms in healthcare workers due to a lack of adequate preparation to deal with the emergency. It is therefore important to intervene early (Lai et al., 2020) with an intervention such as Eye Movement Desensitization and Reprocessing (EMDR) (Shapiro, 2019), as also confirmed by studies conducted in other hospitals (Fogliato et al., 2022).

EMDR therapy is recognized by the World Health Organization (World Health Organization and UNHCR, 2013) as the intervention of first choice in the treatment of disorders of traumatic origin. The EMDR-Integrative Group Treatment Protocol for Ongoing Traumatic Stress (Pérez et al., 2020) uses the Butterfly Hug (Jarero and Artigas, 2014) as a self-administered bilateral stimulation method to reprocess traumatic material.

It was therefore proposed that interested practitioners participate in small group sessions of Brief EMDR.

The 2 fundamental objectives of Brief EMDR work can be summarized as follows:

- Review all traumatic experiences to reduce posttraumatic stress symptoms and prevent dysfunctional processing from leading in the medium and long term to the development of Post Traumatic Stress Disorder (PTSD).
- Working on post-traumatic growth from a traumatic experience.

The aim of this study is, therefore, to test the effectiveness of the EMDR group intervention on a sample of healthcare workers during the COVID-19 health emergency by comparing the pre- and post-treatment phase, the post-treatment phase and follow-up and the pre- and follow-up phase. For this purpose, several self-assessment questionnaires were administered.

## Materials and methods

### Setting

Group meetings were on *de visu* modalities, in compliance with safety regulations, in a large, constantly ventilated room within the Dialysis Service, at a distance, and equipped with Personal protective equipment (PPE).

### Sample

Health care facilities informed all healthcare personnel of the possibility of EMDR intervention to manage the psychological distress caused by the emergency. Freely, facility health workers decided whether or not to take part in the study.

The sample consisted of 43 health workers from the various services: ward, Bassini dialysis and CAL dialysis.

More specifically, the following took part in the research-intervention: 4 nurses and 3 Registered nurses (RNs) from the Nephrology Department, 17 nurses and 1 RN from the Dialysis Service, 8 nurses and 1 RN from the CAL, the nursing coordinator and 8 doctors from the Operating Unit (including the FF Director).

The characteristics of the sample are described in Table 1. The gender (36 female and 7 male), does not appear in the table.

**Table 1:** Socio-demographic characteristics of the sample.

Variable	*N*	Mean	SD
Age	43	48.40	8.02
Schooling	43	2.33	1.04
Children	43	1.35	0.95
No. cohabitants	43	1.93	1.06
Insulation	43	0.39	0.49
Family	43	0.88	0.32

In the follow-up phase, it was possible to reach 36 of the 43 operators previously involved.

### Procedure

Given the mode of operation of the health care providers and the humanitarian aim of the intervention, it was not possible to implement a randomized, delayed treatment condition. Here it is necessary to focus attention on the importance of a prompt intervention vs. a rigorous and well-planned research design.

The study has a follow-up design in that the questionnaires were administered before (PRE), at the end of the intervention (POST) and after 6 months (FU). The first assessment took place for all participants at the beginning of the first meeting while the second at the end of the intervention (after 1 month) and the follow-up assessment after 6 months after the end of intervention.

Clinicians were responsible for pre-post and follow-up assessments but data were collected and analyzed anonymously by other researchers who were doing the data analysis, in this way outcome assessor was masked.

Each participant has read and signed the informed consent and the privacy policy. Once treatment was allowed, subjects had the freedom to leave the study and psychological support at any time. Data were collected anonymously.

### Assessment

The intervention was proposed by the psychologist of the S.C. of Nephrology and Dialysis, trained in the use of the EMDR method and supervised by the EMDR Italy Association.

The composition of the groups was the prerogative of the operators, with a view to the criterion of affinity and taking into account the Services of origin (Ward, Dialysis, CAL). This led to the formation of groups composed as follows:

- Doctors only
- Doctors, Chief and Head Nurse
- Nurses and doctors
- Nurses only
- Nurses and RNs

The choice of the group dimension was dictated by the need to quickly reach as many operators as possible, in order to guarantee a timely intervention, but also by the importance of structuring a work that could give value to the team in its aspects of a resource on a personal and working level. The groups consisted of 2 to 5 operators. A total of 12 groups were activated.

Meetings were organized at times compatible with staff turnover, preferably at the end of working hours.

Each participant signed appropriate consent to participate in the research-intervention, after being informed about privacy aspects.

All participants were administered a socio-demographic questionnaires containing personal data (name, age) and information on their professional qualification, their department of origin before the start of the COVID emergency, employment in a ‘dirty’ or ‘clean’ work area, household composition, living arrangements during the health emergency (isolation or not), the presence of minor children and their location, whether they had contracted the virus, whether they had COVID-positive relatives who had died or not.

Before the start of the EMDR group intervention healthcare workers were asked to fill out two questionnaires: the Emotion Thermometer and the Impact Event Scale- Revised (IES-R).

The Emotion Thermometer is a self-administered questionnaire depicting thermometers, with values between 0 and 10, used to assign a level of intensity to the following emotions or disorders, experienced in the last week: stress, anxiety, depressed mood, anger, sleep disturbance. Finally, there is a sixth column in which to indicate how much help you feel you need to manage these issues.

The IES-R Scale (Weiss and Marmar, 1997) is a self-administered test useful for investigating the presence of post-traumatic symptomatology. It consists of 22 items answered on a Likert scale from 0 (not at all) to 4 (extremely). It consists of 3 subscales (intrusion, avoidance and hyperarousal). It too refers to what was experienced in the week prior to completion. The cut-off for the risk of developing PTSD symptoms is a score of 33 or higher.

At the end of the EMDR treatment the participants filled out the above-mentioned scales again, to which the Post-Traumatic Growth Scale and the Pathway Satisfaction Scale (questionnaire expressing satisfaction or dissatisfaction with the pathway undertaken) were added.

The Post-Traumatic Growth Scale (Tedeschi and Calhoun, 1996), is a self-administered questionnaire containing statements concerning personal changes that occurred following a traumatic event. For each statement, the subject must indicate on a grid a response between “no change” and “very important change.”

With the onset of the second wave of the COVID emergency, the Nephrology Department was again converted into COVID Medicine, and subsequently into the only NO COVID Internal Medicine Department within the hospital. The two Dialysis Services necessarily remained operational to provide the necessary care for COVID and NO COVID dialyzed patients.

It was at this stage, in which retraumatization was underway due to the new pandemic wave, that follow-up questionnaires were administered, with the aim of verifying whether the intervention enabled the new pandemic wave to be tackled with more resources (McAlonan et al., 2007). The tests administered were the IES-R scale and the Emotion Thermometer. Some healthcare workers also completed the Post-Traumatic Growth Scale again.

### Treatment

The Brief EMDR intervention was structured in the following way: 3 face-to-face meetings, with a distance of about 1 week between the first and second intervention and about 2 weeks between the second and third (a total of about 3 weeks from start to closure).

The intervention was implemented at the end of the first wave through the use of a short EMDR group protocol adapted to the COVID emergency, which involves self-administered bilateral stimulation through the Butterfly Hug (Jarero and Artigas, 2014). A significant part of the treatment was also devoted to the installation of resources. More specifically, after a brief psycho-education on the EMDR approach and an agreement on the protection of privacy and respect for speaking turns, the practitioners were asked to recount their experience of the COVID emergency from the first time they heard about Coronavirus until that day. The identification of the most disturbing moment then made it possible to set up the Brief EMDR work with self-stimulation. In the following meetings, the narrative part was devoted to what had happened since the last meeting with regard to the COVID experience and concerns for the future. The Brief EMDR protocol was then applied to the most disturbing moment. In all group sessions, sample space was devoted to stabilization techniques (such as grounding) and the installation of resources, e.g., with the use of the ‘I have, I am, I can’ card. Although in most situations, long and individual therapy, this might not be a problem, there are conditions in which many people must be treated at the same time, such as after natural or man-made disasters or as in the earlier described context (COVID emergency). In such cases, and with limited resources, EMDR group might be an initial solution of choice.

So, the EMDR Integrative Group Treatment Protocol (IGTP) has achieved good results and has been widely used in many countries (Jarero et al., 2014). It has been shown to be beneficial in large-scale disaster situations. This protocol is also variously known as the Group Butterfly Hug Protocol, The EMDR Group Protocol, and the Brief EMDR protocol was adapted to the COVID emergency as shorter and more ecologically applicable.

The protocol applied during the intervention:

First meeting:

1. *Narrative,* narrative of the COVID experience from its origins to the time of the beginning of intervention.
2. *Reprocessing* with bilateral stimulation through the butterfly hug.
3. *Stabilization* with Grounding exercise, preferred to breathing exercises to avoid placing too much emphasis on the very breath (immediately associated by some participants with the COVID patient).
4. *Installation* of resources with a reflection on the strength that has helped so far.
5. *Dust exercise*.

Second meeting:

1. *Narrative*, telling of what happened during the past week.
2. *Reprocessing* with bilateral stimulation through the butterfly hug.
3. *Resource* work through the “I have, I am, I can” exercise to install positive perceptions with respect to oneself during the COVID experience.
4. *Stabilization* exercise.
5. *Dust exercise.*

Third meeting:

1. *Narrative*, narrative of what happened during the past 2 weeks (from the last meeting to the current one).
2. *Reprocessing* with bilateral stimulation through butterfly hugging.
3. *Post-traumatic growth* work with a request to represent on a blank sheet of paper (in written or figurative form) what you feel you learned positive about yourself from the COVID experience.
4. *Installation* of identified resources.
5. *Dust exercise.*

### Statistics

The whole data analysis is based on three comparisons: delta1 = difference between pre and post situation, delta2 = difference between situation post and follow-up (FU) situation, delta 3 = difference between pre and FU situation.

These three comparisons correspond to the initial treatment effect (delta1), decreasing treatment effect over time (delta2), to the maintenance of a better situation than the initial situation over long times (delta3). For homogeneity and simplicity of interpretation then, numerically delta1 = PRE - POST, delta2 = FU - POST, delta3 = PRE - FU.

Of course, having paired data (i.e., on the individual subject) makes it possible to calculate delta values for all the descriptors considered at the level of the individual.

Demographic variables (age, schooling, children, conviviality, isolation, family) were transformed into variables by rank (whereby schooling ranges from 1 to 4 following the order, high school diploma, postgraduate course, bachelor’s degree, master’s degree), the condition of isolation and family life follow the classic pattern 1 = yes, 0 = no, so the mean of these variables corresponds to the proportion of subjects answering ‘yes,’ while gender [mostly female (36 F and 7 M)] does not appear in this summary table.

The PTGI variables deserve a separate discussion: they are already ‘differential’ respectively of greater awareness acquired in the period between PRE and POST and in that between POST and FU, since these variables can only take positive values (the measurement scheme does not provide for ‘arrears’) and checked the strong correlation between the values of different PTGI descriptors, the difference between the first period (PRE-POST) and the second period (FU) as the difference deltaptgi = ptgitotalPOST - ptgitotalPRE. In this case, positive values significantly different from zero of deltaptgi indicate a weakening of the beneficial effect over time or, at least a slowing down of the improvement.

Inferential statistics on the delta variables testing the null hypothesis of delta = 0, equivalent to a paired test paradigm, was computed by means of non-parametric (Sign and SignedRank) and parametric (t-test) approach. The statistical significance was estimated by a non-parametric approach based on Wilcoxon scores evaluated by a chi-square Kruskal-Wallis test.

## Results

The main socio-demographic characteristics of the sample are summarized in Table 1. The gender, mostly female (36 F and 7 M), does not appear in the table.

Table 2 summarizes the PRE, POST and FU analyses concerning the 3 subscales of the IES-R questionnaire, i.e., Intrusion, Avoidance and Hyperarousal, and the 6 items of the Emotion Thermometer.

**Table 2:** PRE, POST and FU analyses.

Variable	*N*	M pre	SD pre	*N*	M post	SD Post	*N*	M F-U	SD F-U
IESAVOIDANCE	43	14.12	5.56	43	10.14	8.40	43	10.33	6.55
IESINTRUSIVENESS	43	16.14	7.05	43	8.47	6.70	43	10.07	8.01
IESHYPERAROUSAL	43	12.12	5.37	43	5.51	4.93	43	7.67	5.09
IESTOT	43	42.37	15.83	43	24.12	18.53	43	28.07	18.34
Termo1	41	5.71	2.36	43	2.35	1.73	43	4.23	2.49
Termo2	41	5.51	2.64	43	2.14	1.78	43	3.65	2.39
Termo3	40	4.55	2.75	43	1.56	2.03	43	2.72	2.42
Termo4	41	5.05	2.82	43	2.07	2.25	43	3.72	3.25
Termo5	41	5.15	2.96	43	1.81	2.35	43	3.56	3.26
Termo6	41	5.10	2.57	42	1.95	1.85	*42*	3.02	2.92

As can be seen, the mean total values on the IES-R scale administered before treatment indicate a high risk for the healthcare workers to develop PTSD symptoms. On the other hand, the data collected post-treatment and at follow-up show mean total values on the IES-R scale of less than 33, and thus no risk of PTSD.

Looking at the values for the 3 subscales of the IES-R scale and the total, it is evident that they tend to increase in the follow-up phase compared to the post-treatment phase.

With regard to the data obtained in the different items of the Emotion Thermometer, it is also possible to observe an important decrease in values in the post and follow-up phase compared to the pre-treatment phase.

In order to better understand the variations described above, it is necessary to compare the data collected pre-, post and f-u, i.e., to analyze the various DELTAs (Table 3). More specifically, DELTA 1 concerns the difference between pre and post situation, DELTA 2 between post and follow-up, and DELTA 3 between pre and follow-up situation.

**Table 3:** Analysis of DELTAs.

Variable	M DELTA1	SD DELTA1	M DELTA2	SD DELTA2	M DELTA3	SD DELTA3	*p*
DELTA avoid	3.98	7.68	0.19	8.60	3.79	6.42	<0.001
DELTA intrus	7.67	6.15	1.60	6.55	6.07	6.20	<0.001
DELTA hyper	6.60	4.46	2.16	4.76	4.44	4.29	<0.001
DELTA tot	18.26	15.84	3.95	17.99	14.30	14.02	<0.001
DELTA termo1	3.32	2.39	1.88	2.58	1.34	2.74	<0.001
DELTA termo2	3.32	2.15	1.51	2.36	1.69	2.62	<0.001
DELTA termo3	2.95	2.60	1.16	2.40	1.72	2.85	<0.001
DELTA termo4	3.05	2.73	1.65	3.52	1.17	3.43	<0.001
DELTA termo5	3.24	2.76	1.74	3.41	1.41	3.15	<0.001
DELTA termo6	3.10	2.56	1.10	2.58	2.00	3.09	<0.001

Student’s *t* test and non-parametric tests performed on the DELTA values allow the statistical significance of the various comparisons to be verified.

DELTA1 analysis of the 3 subscales of the IES-R highlights the change that occurred between the pre- and post-treatment phase in terms of symptom reduction. All DELTA1 variables, both related to the IES and the Emotion Thermometer, show a significant improvement in terms of both parametric (Student’s t test) and non-parametric (Signs and Ranks with sign) tests.

All DELTA2 variables, except for avoidance, show a significant weakening of the effect with time, but the magnitude of this effect is much smaller than the improvement found in DELTA1 (as evidenced by the significantly lower averages of DELTA2 values compared to DELTA1).

On the basis of these premises, statistical significance being highlighted, it can in fact be observed that the magnitude of the waning of the treatment effects (DELTA2) is in any case less than the maintenance (DELTA3).

If we focus on the values of the DELTA Totals, we see that the average value of DELTA1 is about 18, that of DELTA2 about 4, that of DELTA3 about 14, so it can be said that about 22% of what was gained was lost in the follow-up.

**Figure 1:**
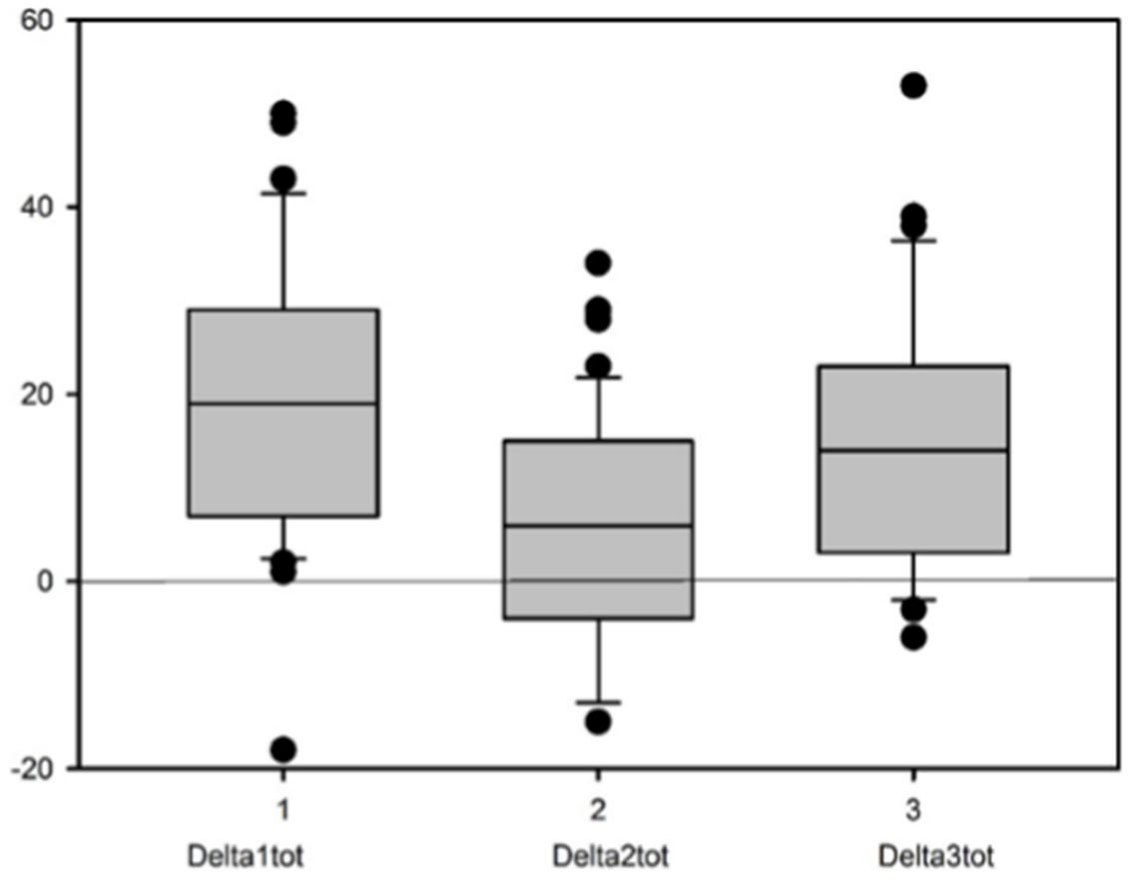
Values of DELTAtot.

The box plots shown in Figure 1 refer to the values of DELTAtot. It can be seen that the fading of the initial effect (DELTA2tot) is rather close to the zero line (no difference) while both the initial effect (DELTA1tot) and the long-term residual effect (DELTA3tot) deviate.

There was no significant effect of demographic variables on DELTA values, with the exception of gender showing a weak significant effect only on DELTA1.

**Figure 2:**
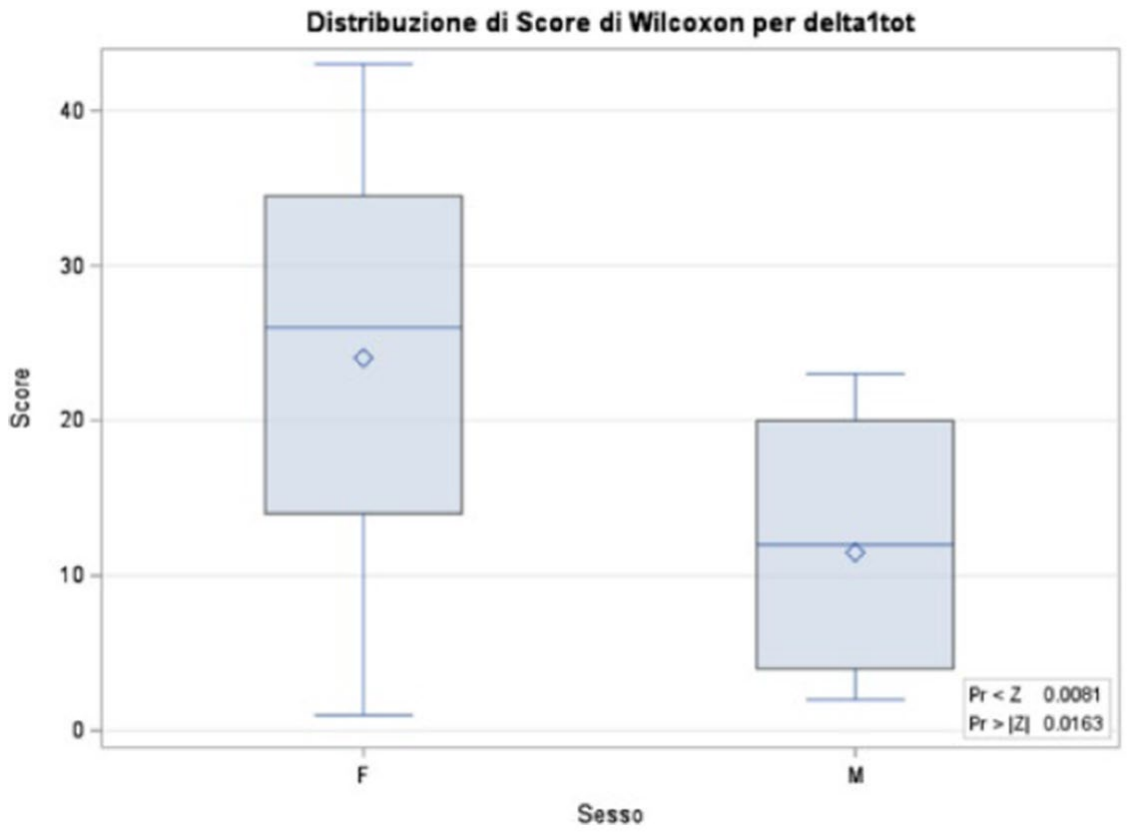
Analysis with Wilcoxon’s 2-sample test.

Analysis with Wilcoxon’s 2-sample test reveals a Wilcoxon score distribution for DELTA1tot shown in Figure 2.

Table 4 summarizes the values for the Post-Traumatic Growth Scale and DELTA analysis.

**Table 4:** Summarizes the values for the Post-Traumatic Growth Scale and DELTA analysis.

Variable	M POST	SD POST	M F-U	SD F-U
ReportPTGI	25.98	8.53	18.42	9.08
PossiblePTGI	26.98	8.53	14.06	5.82
ForzaPTGI	17.05	4.82	12.81	5.29
SpiritoPTGI	5.95	3.21	4.72	2.98
AppreciatePTGI	12.19	3.97	11.50	3.59
PTGItotal	78.88	23.04	61.50	23.27
DELTAPTGI	M 17.61	DS 20.32		

The PTGI variables are already ‘differentials’ respectively of increased awareness in the period between PRE and POST and in the period between POST and FU, as these variables can only take on positive values. Focusing on the strong correlation, through Spearman’s rank test, between the values of the different PTGI descriptors, the difference between the first period (PRE-POST) and the second period (FU) was summarized as DELTAptgi = ptgitotalPOST - ptgitotalPRE.

Positive values significantly different from zero of DELTAPTGI indicate a weakening of the beneficial effect over time or, at least, a slowing down of the improvement.

The DELTA between the PTGItotal measured in the POST condition and that measured in the FU condition illustrates the difference between the gain in awareness obtained in the phase between PRE and POST, compared to that obtained during the Follow-up. The PTGI value between PRE and POST is significantly higher than the gain in awareness obtained in the follow-up.

The Satisfaction Scale administered to the participants at the end of the course highlighted their satisfaction and sense of effectiveness. It should be emphasized that all 43 practitioners would recommend the EMDR support to their colleagues.

## Discussion

The aim of this study was to assess the effectiveness on of the Brief EMDR group treatment in reducing PTSD symptoms proposed to healthcare workers and the maintenance over time of the results achieved, by comparing the data collected before treatment (PRE), after treatment (POST) and in the follow-up phase (FU). The comparison between PRE and POST makes it possible to assess the effectiveness immediately after treatment; the comparison between POST and FU highlights the possible weakening of the results obtained after time, in a phase of re-traumatization of the traumatic event, i.e., re-traumatization during the second and third wave; the comparison between PRE and FU makes it possible to assess the maintenance of results over time.

A further objective was to measure post-traumatic growth at post-treatment and follow-up, and to compare them.

The mean total values on the IES-R scale administered before treatment indicate a high risk for the caregivers to develop posttraumatic stress symptoms. On the other hand, the data collected post-treatment and at follow-up show mean total values on the IES-R scale of less than 33, and therefore no risk of posttraumatic stress symptoms.

The comparison between PRE and POST treatment (DELTA1) regarding the 3 subscales of the IES-R, highlighted the important change that occurred in terms of symptomatology reduction. More specifically, all DELTA1 variables, both related to the IES and the Emotion Thermometer, showed a significant improvement.

By comparing POST and FU (DELTA2), it was observed that all variables except avoidance show a significant weakening of the effect with time, but the magnitude of this effect is much smaller than the improvement found in DELTA1.

DELTA 3 analysis finally made it possible to highlight how the treatment effect, although weakened, is maintained almost intact at follow-up. In fact, the maintenance of a better situation at follow-up was observed, in the course of re-traumatization linked to the new wave, compared to the initial data.

In summary, the initial beneficial effect of the treatment (DELTA 1), the decrease of the treatment effect over time (DELTA 2) and the maintenance of a better situation than the initial situation over a longer period of time (DELTA 3) were highlighted.

As previously described, there was no significant effect of demographic variables on DELTA values, with the exception of gender, which only showed a weak significant effect on DELTA1, but the large difference in the numerosity of the two groups makes this result unreliable.

With regard to the analysis of post-traumatic growth, through the PTGI questionnaire, an increase in awareness was observed in the phase between PRE and POST. It was also observed, from the analysis of the questionnaire administered in the FU phase and from the comparison with the previous one, a weakening of the beneficial effect over time or, at least, a slowing down of the improvement.

## Limits

The main limitation of this study is the absence of a control group. A limited number of practitioners, who decided not to participate in the EMDR treatment, filled in the questionnaires at an early stage and after a few months. As this was a very small group (7 persons), it was precisely not possible to consider it a control group.

Another limitation is represented by the spontaneous sampling. The recruitment of the subjects, which took place by free choice, in fact represents a self-selection bias, making the participants a sample that is not fully representative of the entire category of healthcare workers.

## Conclusion

The research-intervention demonstrates the effectiveness of EMDR treatment and its progress over time. More specifically, the significant clinical improvement following group treatment with Brief EMDR has emerged. The extent of the waning of the treatment effects is, however, less than maintenance. At the follow-up, which took place in correspondence with a re-traumatization of the operators due to the new pandemic wave, the maintenance of a better situation than at the beginning was observed. This allowed the healthcare workers subjected to the intervention to cope with the new pandemic wave with more tools. The new stress factors certainly affected the mental health of the staff, but the values recorded in the questionnaires administered were not indicative of the risk of developing PTSD.

Those who gained more benefit in the first phase also lost more in the second, but never reached the pre-treatment disturbance levels.

The raw data supporting the conclusions of this article are made available by the authors.

The participants provided their written informed consent to participate in this research-intervention.

## Data availability statement

The raw data supporting the conclusions of this article will be made available by the authors, without undue reservation.

## Ethics statement

Ethical review and approval was not required for the study on human participants in accordance with the local legislation and institutional requirements. The patients/participants provided their written informed consent to participate in this study.

## Author contributions

CB contributed to conception, design, realization of the study, and organized the database. GM and IF contributed to conception and design of the study. MP performed the statistical analysis. EG contributed to translate the study. PF, EA, and SG wrote sections of the manuscript. All authors contributed to manuscript revision, read, and approved the submitted version.

## Conflict of interest

The authors state that the research was conducted in the absence of commercial or financial relationships that could be interpreted as a potential conflict of interest.

## Publisher’s note

All claims expressed in this article are solely those of the authors and do not necessarily represent those of their affiliated organizations, or those of the publisher, the editors and the reviewers. Any product that may be evaluated in this article, or claim that may be made by its manufacturer, is not guaranteed or endorsed by the publisher.

## References

[ref001] Brady J. L. Guy J. D. Poelstra P. L. Brokaw B. F. (1999). Vicarious traumatization, spirituality, and the treatment of sexual abuse survivors: A national survey of women psychotherapists. Prof. Psychol. Res. Pract. 30, 386–393. doi: 10.1037/0735-7028.30.4.386

[ref2] Campion J. Javed A. Sartorius N. Marmot M. (2020). Addressing the public mental health challenge of COVID-19. Lancet Psychiatry 7, 657–659. doi: 10.1016/S2215-0366(20)30240-6, PMID: 32531299 PMC7282758

[ref4] Chan A. O. M. (2004). Psychological impact of the 2003 severe acute respiratory syndrome outbreak on health care workers in a medium size regional general hospital in Singapore. Occup. Med. 54, 190–196. doi: 10.1093/occmed/kqh027, PMID: 15133143 PMC7107861

[ref5] Dyregorov A. Yule W. (2008). Psychological interventions in disasters – reflections from professional experience. Tidsskrift for norsk psykologforening 45, 1512–1516. doi: 10.1080/02682620808657710

[ref7] Fogliato E. Invernizzi R. Maslovaric G. Fernandez I. Rigamonti V. Lora A.. (2022). Promoting mental health in healthcare Workers in Hospitals through Psychological Group Support with eye Movement Desensitization and Reprocessing during COVID-19 pandemic: an observational study. Front. Psychol. 12:794178. doi: 10.3389/fpsyg.2021.794178, PMID: 35153919 PMC8829464

[ref8] Giusti E. M. Pedroli E. D’Aniello G. E. Stramba Badiale C. Pietrabissa G. Manna C.. (2020). The psychological impact of the COVID-19 outbreak on health professionals: a cross-sectional study. Front. Psychol. 11:1684. doi: 10.3389/fpsyg.2020.01684, PMID: 32754102 PMC7366071

[ref9] Iacolino C. Cervellione B. (2019). Gli operatori dell’emergenza: Fattori di rischio e di protezione. Milan, Italy: Franco Angeli.

[ref1] Jarero I. Artigas L. (2014). “The EMDR integrative group treatment protocol (IGTP) for adults,” in Implementing EMDR Early Mental Health Interventions for Man-Made and Natural Disasters. ed. Luber M. (New York, NY: Springer), 253–265.

[ref003] Jarero I. Artigas L. Uribe S. Miranda A. (2014). EMDR therapy humanitarian trauma recovery interventions in Latin America and the Caribbean. J. EMDR Pract. Res. 8, 260–268. doi: 10.1891/1933-3196.8.4.260

[ref11] Lai J. Ma S. Wang Y. Cai Z. Hu J. Wei N.. (2020). Factors associated with mental health outcomes among health care workers exposed to coronavirus disease 2019. JAMA Netw. Open 3:e203976. doi: 10.1001/jamanetworkopen.2020.3976, PMID: 32202646 PMC7090843

[ref12] Lee S. M. Kang W. S. Cho A.-R. Kim T. Park J. K. (2018). Psychological impact of the 2015 MERS outbreak on hospital workers and quarantined hemodialysis patients. Compr. Psychiatry 87, 123–127. doi: 10.1016/j.comppsych.2018.10.003, PMID: 30343247 PMC7094631

[ref004] Lee A. M. Wong J. G. McAlonan G. M. Cheung V. Cheung C. Sham P. C.. (2007). Stress and psychological distress among SARS survivors 1 year after the outbreak. Can. J. Psychiatry. Revue canadienne de psychiatrie 52, 233–240. doi: 10.1177/070674370705200405 17500304

[ref13] Lin C.-Y. Peng Y.-C. Wu Y.-H. Chang J. Chan C.-H. Yang D.-Y. (2007). The psychological effect of severe acute respiratory syndrome on emergency department staff. Emerg. Med. J. 24, 12–17. doi: 10.1136/emj.2006.035089, PMID: 17183035 PMC2658141

[ref15] Matsuishi K. Kawazoe A. Imai H. Ito A. Mouri K. Kitamura N.. (2012). Psychological impact of the pandemic (H1N1) 2009 on general hospital workers in Kobe: pandemic in Kobe. Psychiatry Clin. Neurosci. 66, 353–360. doi: 10.1111/j.1440-1819.2012.02336.x, PMID: 22624741

[ref16] McAlonan G. M. Lee A. M. Cheung V. Cheung C. Tsang K. W. Sham P. C.. (2007). Immediate and sustained psychological impact of an emerging infectious disease outbreak on health care workers. Can. J. Psychiatry 52, 241–247. doi: 10.1177/070674370705200406, PMID: 17500305

[ref18] Pérez M. C. Estévez M. E. Becker Y. Osorio A. Jarero I. Givaudan M. (2020). Multisite randomized controlled trial on the provision of the EMDR integrative group treatment protocol for ongoing traumatic stress remote to healthcare professionals working in hospitals during the Covid-19 pandemic. Psychol. Behav. Sci. Int. J. 15, 1–12. doi: 10.19080/PBSIJ.2019.10.555920

[ref22] Shapiro F. (2019). EMDR: Il manuale: principi fondamentali, protocolli e procedure. Nuova Edn R. Cortina. (Milano).

[ref005] Sinclair H. A. Hamill C. (2007). Does vicarious traumatisation affect oncology nurses? A literature review. Eur. J. Oncol. Nurs. 11, 348–356. doi: 10.1016/j.ejon.2007.02.007 17482879

[ref23] Taylor A.S.W. Frazer A. G. (1981). Psychological sequelae of operation overdue following the DC 10 aircrash in Antartica. Wellington: Victoria University of Wellington, 72.

[ref25] Tedeschi R. G. Calhoun L. G. (1996). The posttraumatic growth inventory: measuring the positive legacy of trauma. J. Trauma. Stress. 9, 455–471. doi: 10.1007/BF02103658, PMID: 8827649

[ref26] Weiss D. S. Marmar C. R. (1997). “The impact of event scale-revised” in Assessing psychological trauma and PTSD. eds. Wilson J. P. Keane T. M. (Guilford Press), 187–198.

[ref27] World Health Organization (2020). Coronavirus disease (COVID-19) outbreak: rights, roles and responsibilities of health workers, including key considerations for occupational safety and health. Geneva: World Health Organization.

[ref28] World Health Organization & UNHCR (2013). “Assessment and management of conditions specifically related to stress: MhGAP intervention guide mode” in Evaluación y manejo de condiciones específicamente relacionadas con el estrés: Módulo de la guía de intervención mhGAP (Geneva: World Health Organization)

